# Hybrid Approach: Combining Surgical Thrombectomy and AngioJet™ Aspirational Thrombectomy in Limb Graft Occlusion Post-FEVAR with Fenestrated Anaconda™ and in ePTFE Bypass Graft Occlusion

**DOI:** 10.3390/jcm13144002

**Published:** 2024-07-09

**Authors:** Gowri Kiran Puvvala, Karamperidis Loukas, Konstantinos P. Donas, Juergen Hinkelmann, Ba-Fadhl Faiz, Luna Vidriales Gerado, Anastasios Psyllas

**Affiliations:** 1 Department of Vascular Surgery, Marien Hospital, 46483 Wesel, Germany; 2 Department of Vascular Surgery, Asklepios Clinic, 63225 Langen, Germany

**Keywords:** mechanical thrombectomy, AngioJet™ Ultra Thrombectomy System, occlusion, bypass graft, stent graft, Rutherford classification, Solent™ Proxi, ZelanteDVT™, Iliac limb graft, FEVAR, fontaine, iliopopliteal bypass, ePTFE, DSA, rTPA, POBA, limb graft occlusion (LGO), aspirational thrombectomy, Fogarty thrombectomy

## Abstract

Acute limb ischemia due to limb-graft occlusion (LGO) after fenestrated endovascular aneurysm repair (FEVAR) and acute bypass graft occlusion with an ePTFE graft pose critical challenges, necessitating prompt intervention to prevent limb loss. This paper discusses two cases of acute limb ischemia treated with a hybrid approach using the AngioJet™ Ultra Thrombectomy System as an adjunct to Fogarty thrombectomy. Case I involved a 69-year-old male post-FEVAR with contralateral iliac limb graft occlusion of the fenestrated Anaconda™, while Case II featured a 70-year-old male (ASA IV) post-bypass surgery (iliopopliteal arterial bypass with ePTFE Graft) with acute bypass graft occlusion. Both cases underwent successful recanalization using the AngioJet™ Ultra Thrombectomy System (ZelanteDVT™ 8F catheter, Solent™ Proxi 6F catheter) (Boston Scientific, Marlborough, MA, USA), combined with adjunctive techniques including Fogarty thrombectomy, balloon angioplasty, stenting, and local lysis. Immediate postoperative and follow-up assessments after 6 months revealed restored limb perfusion and improved clinical outcomes, with palpable pulses and improved ulcer healing. The aim of this treatment strategy is not only to alleviate limb ischemia but also to preserve future options in the event of graft failure. The use of the AngioJet™ Thrombectomy System in cases of LGO aims not only to clear the thrombus load but also to avoid the need for graft relining. In the case of acute arterial bypass graft occlusion in a patient with ASA IV, the goal of using the thrombectomy device is to preserve the native vessels for future procedures, such as long infragenual bypass, in addition to limb salvage. These cases demonstrate the efficacy of a hybrid surgical approach in managing acute limb ischemia following graft occlusion following FEVAR and bypass surgery. Long-term follow-up will further elucidate the durability of these interventions and their impact on limb salvage and overall patient outcomes. By combining mechanical thrombectomy with adjunctive techniques, such as balloon angioplasty and stenting, this hybrid approach offers a comprehensive solution to acute limb ischemia, addressing both the underlying occlusive pathology and ensuring optimal limb perfusion. Furthermore, the utilization of the AngioJet™ Ultra Thrombectomy System provides a minimally invasive yet effective method for thrombus removal, reducing procedural time and potential complications associated with open surgical techniques. As such, this approach represents a valuable addition to the armamentarium of treatments for acute limb ischemia, particularly in cases of graft occlusion following complex endovascular and bypass procedures.

## 1. Background and Aim

A hybrid approach, combining Fogarty thrombectomy with additional aspirational thrombectomy using Angiojet™ Ultra Thrombectomy Device, in the treatment of limb graft occlusion (LGO) after FEVAR with a fenestrated Anaconda™ custom-made device, as well as in the management of occlusion of an iliopopliteal arterial bypass (ePTFE graft) and the runoff vessels, has not been reported to date. In this paper, we present the outcomes of two cases wherein successful recanalization of the grafts was achieved using a hybrid approach with the AngioJet™ Ultra Thrombectomy System, a pharmaco-mechanical aspirational thrombectomy device, as an adjunct to Fogarty thrombectomy. 

This device was chosen due to its flexible catheters and over-the-wire system. Moreover, the AngioJet™ thrombectomy device has various suitable catheters that can be deployed in large conduits as well as vessels as small as 1.5 mm, making it versatile for use in different vascular conduits. Additionally, diagnostic angiography can be performed intraoperatively during the procedure over the catheter, and in the case of catheter-directed thrombolysis (CDT), the catheter can be left in situ.

The aim of using the AngioJet™ Thrombectomy System in the case of limb graft occlusion is not only to clear the thrombus load but also to avoid relining if possible. In the case of acute arterial bypass graft occlusion in a patient with ASA IV, the goal of using the thrombectomy device is to preserve the native vessels for future procedures, such as long infragenual bypass, in addition to limb salvage.

## 2. Case Presentations

### 2.1. Case I

**Figure 1:**
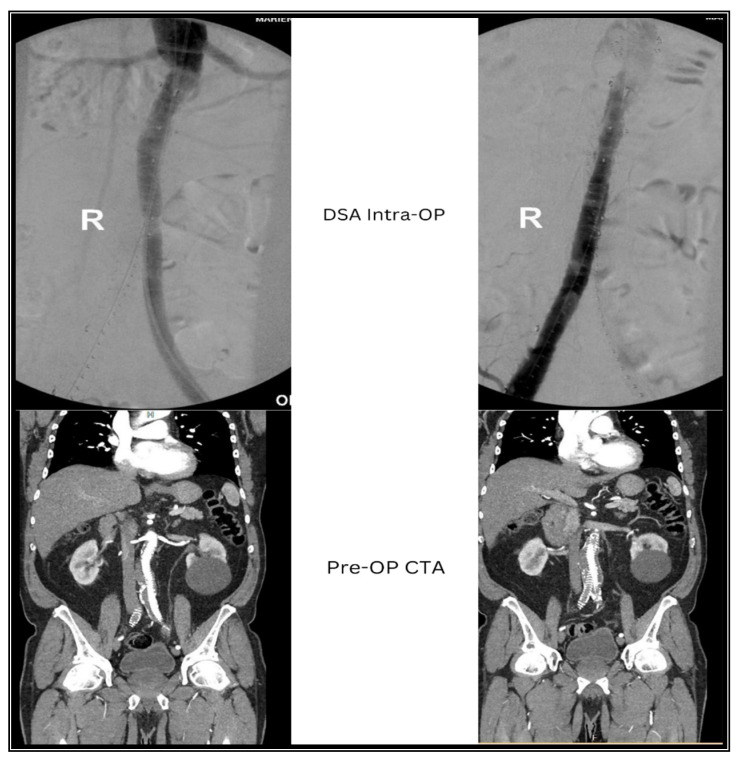
Pre-operative ct-angiography (Pre-OP CTA) and intraoperative digital subtraction angiography (DSA Intra-OP).

A 69-year-old male, ASA III, who underwent FEVAR with a fenestrated Anaconda™ endoprosthesis and 4 bridging stents, presented to our department with symptoms of acute limb ischemia (Fontaine III). Sonographic and radiological assessments revealed occlusion of the contralateral iliac limb graft (right side). The ipsilateral external iliac artery and femoral vessels were open, with an absence of inguinal pulse. Following our planned approach, we performed a Fogarty thrombectomy. Intraoperative subtraction angiography showed residual thrombus and adherent wall thrombus, persistently occluding the proximal end of the iliac limb graft (approximately 4 cm). Further attempts to advance the Fogarty catheter proximal to the occlusion, as well as balloon angioplasty, were unsuccessful (Figure 1).

### 2.2. Case II

**Figure 2:**
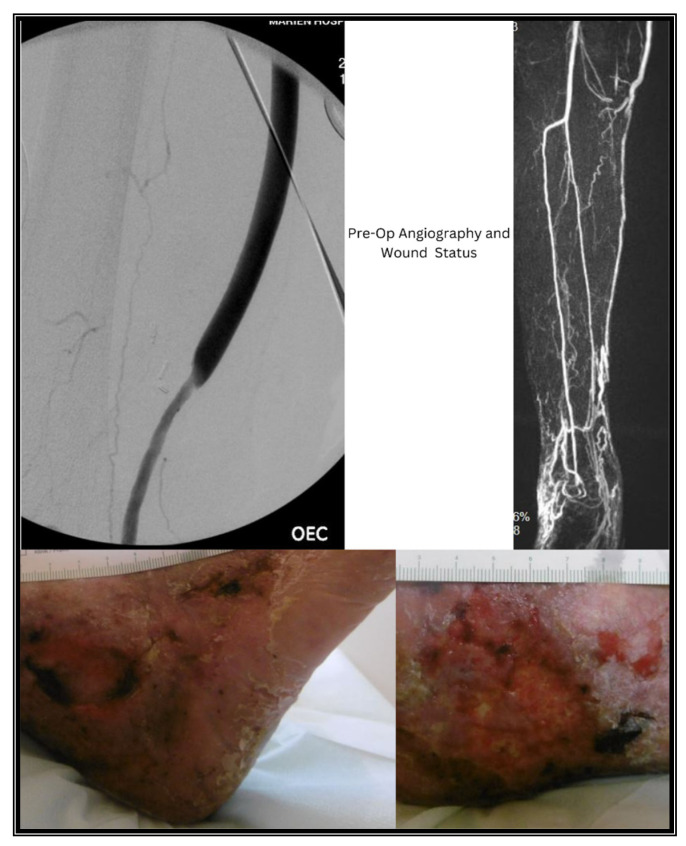
Pre-Operative Wound Status and Angiography.

**Figure 3:**
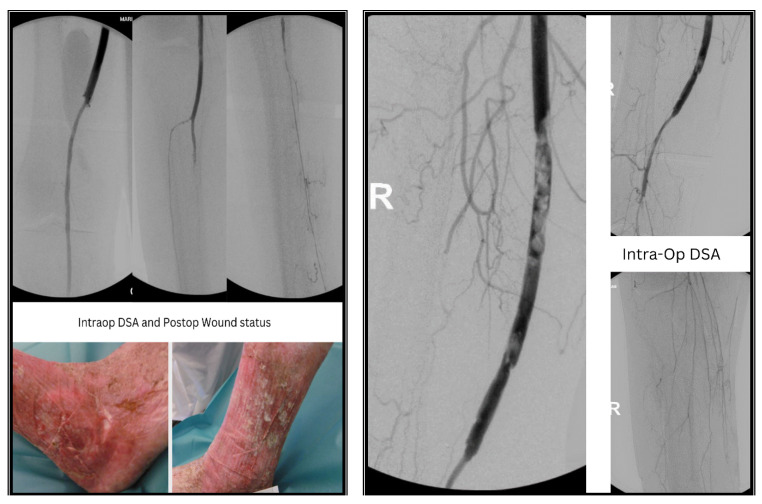
Intra-Operative DSA and postoperative wound status.

A 70-year-old male, ASA (American Society of Anesthesiologists) IV, with a history of multiple vascular surgeries including multiple bypass surgeries and inguinal revisions on the ipsilateral side, recently underwent an iliopopliteal seg. I bypass with an ePTFE graft and prothesio-profunda bypass due to a massive pseudoaneurysm in the ipsilateral groin, critical limb ischemia, and non-healing ulcers (Rutherford category V). The blood flow was restored, and postoperatively, the ulcers began to heal. However, after two weeks, the patient complained of severe rest pain, and the ulcers showed new signs of necrosis (Figure 2). Duplex sonography revealed acute occlusion of the bypass graft. The next day, we performed a Fogarty thrombectomy of the bypass graft, and intraoperative DSA showed complete occlusion of the runoff vessels distal to the bypass (Figure 3).

## 3. Methods & Results

### 3.1. Case I

In this scenario, following Fogarty thrombectomy, multiple balloon angioplasties, and intraoperative angiography, residual thrombus and adherent wall thrombus persisted, rendering all attempts to recanalize the iliac limb graft with a Fogarty catheter unsuccessful. To restore perfusion, the decision was made to utilize the AngioJet™ Thrombectomy System intraoperatively. We employed the ZelanteDVT™ (Boston Scientific, Marlborough, MA, USA) 8F aspiration catheter and successfully aspirated the residual thrombus within a device runtime of 300 s. Subsequently, the procedure was augmented with plain old balloon angioplasty (POBA), intraoperative thrombolysis with recombinant tissue plasminogen activators (rTPA), and local desobliteration and patch angioplasty of the ipsilateral femoral vessels.

**Figure 4:**
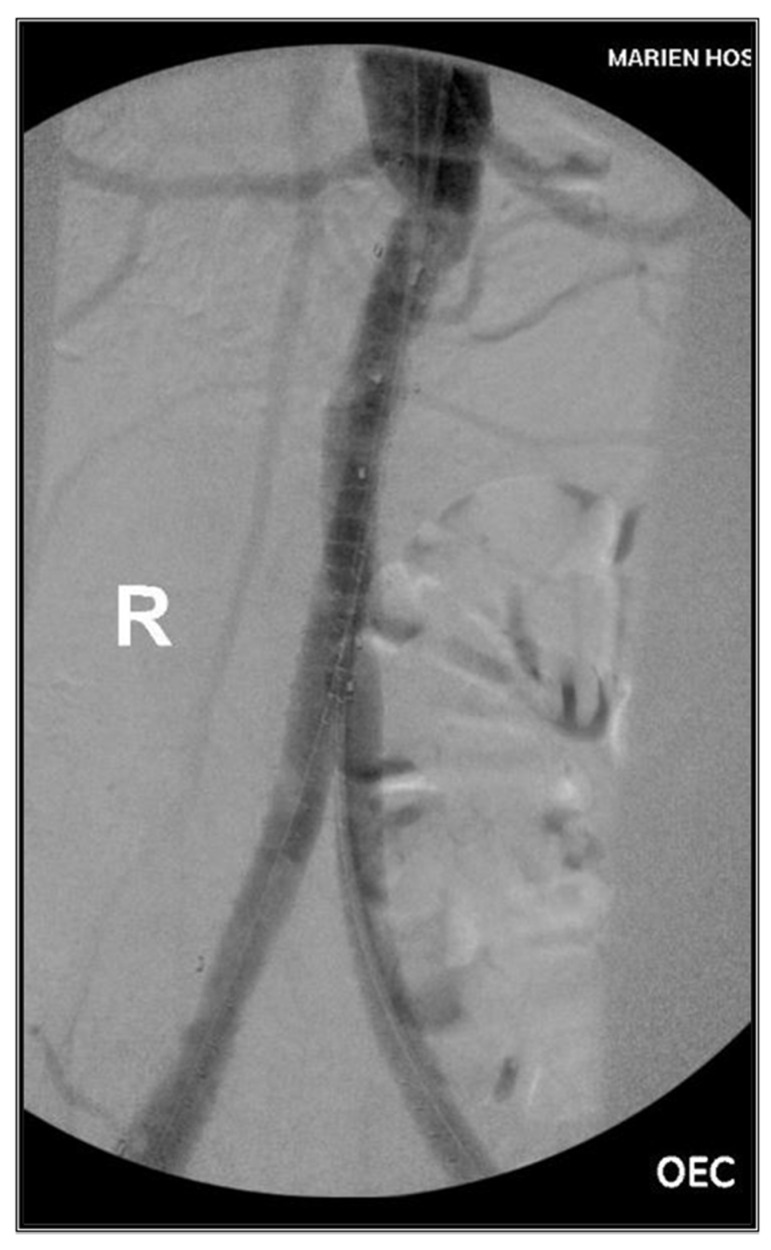
End DSA.

Upon completion, intraoperative subtractions angiography revealed successful recanalization of the iliac limb graft and the peripheral vessels, with no residual stenosis and no signs of stent dislocation or endoleak. Following the successful intervention, the patient experienced an immediate regression of symptoms. Both immediately postoperatively, 2 months and 6 months later, palpable groin and popliteal pulses were evident. Sonographically, both the iliac limb graft and the femoral vessel demonstrated triphasic perfusion. Radiologically, there were no signs of endoleak, graft dislocation, or displacement of the bridging stents (Figure 4).

### 3.2. Case II

After initial attempts with Fogarty thrombectomy revealed a persisting thrombus load and complete occlusion of the runoff vessel distal to the bypass (Figure 3), considering the patient’s ill condition, we opted for a hybrid endovascular approach using the AngioJet™ Ultra Thrombectomy System (Boston Scientific, Marlborough, MA, USA) with a Solent™ Proxi 6F catheter. The aspirational thrombectomy device aspirated the remaining thrombus from the distal anastomosis, popliteal artery, and runoff vessels within 300 s. Control DSA revealed dissection at the distal anastomosis and popliteal artery Segment I (distal to the anastomosis). We subsequently combined the procedure with angioplasty with drug-coated-balloon (DCB) and stenting of the distal anastomosis and popliteal artery Segment I using a self-expanding nitinol stent. Due to the persisting thrombus load, local lysis was performed intraoperatively using Actilyse^®^ (rTPA). Finally, the entry site of the bypass was treated with bovine patch angioplasty.

Post mechanical aspirational thrombectomy, stenting, Angioplasty with drug coated balloon and local lysis showed angiographically successful perfusion of the tibial vessels, with no signs of residual stenosis or dissections. Immediately postoperative, 2 months and 6 months later, groin and popliteal pulses were palpable. The ulcers showed dramatic improvement in terms of healing. Sonographically, the bypass graft and the popliteal artery demonstrated biphasic perfusion, while both the tibial vessels showed mono-biphasic perfusion (Figure 3).

In both cases, patients experienced transient, self-limiting hemoglobinuria. At the time of writing the article, sonographic patency of the grafts was maintained for 6 months. Further follow-up will determine the long-term success of these hybrid interventions.

## 4. Discussion

In the case of iliac limb graft occlusion, we deployed the ZelanteDVT™ (Boston Scientific, Marlborough, MA, USA) 8F catheter due to the large diameter of the iliac limb and the thrombus load. The ZelanteDVT™ 8F catheter can be used in vessels with a diameter ≥ 6 mm, has a working length of 105 cm, and a maximum total run time of 480 s. The catheter requires an 8 F sheath. While working with this catheter, one must concentrate on the maximum device run time to prevent device-related complications.

For the ePTFE (6 mm) bypass graft occlusion, we used the Solent™ Proxi 6F catheter. As antegrade aspiration thrombectomy of the tibial vessels and the bypass graft would suffice for the minimum diameter prescribed and to prevent dissection as well as perforation of the vessel. The Solent™ Proxi (Boston Scientific, USA) 6F catheter can be utilized in vessels with a minimum diameter of 3 mm, has a working length of 90 cm, and has a maximum total run time of 480 s. Even after following necessary precautions, due to the usage of the Fogarty catheter at the distal bypass anastomosis and the popliteal artery, a dissection developed. Hence, a self-expanding stent application was chosen. In both cases, the maximum total run time was 300 s. 

Acute limb ischemia with limb graft occlusion (LGO) following EVAR or FEVAR is a critical complication, with an incidence ranging from 2.6–7.4% [1]. The endograft device type is an independent risk factor for LGO after EVAR. Specifically, the Zenith Alpha demonstrated an increased risk of LGO compared with the Endurant and Excluder devices. The strongest predictive factors of LGO include extension in the external iliac artery, small diameter (diameter ≤ 10 mm), tortuous, angled, and calcific iliac axis, excessive oversizing of the limb graft, kinking, use of old-generation devices, and EVAR performed outside the instructions for use. Limb graft oversizing >15%, kinking, and aortic bifurcation < 20 mm appears to be independent predictive factors of LGO.

According to the literature, some documented cases with limb graft occlusion after implantation of fenestrated anaconda endoprosthesis exist, but the treatment approach for LGO in fenestrated endografts has not been conceptualized. The dynamics in terms of LGO after FEVAR/BEVAR, in comparison to LGO after EVAR, vary dramatically, as the presence of bridging stents or branching stents may limit treatment options and present challenges.

Current general treatments for limb graft occlusion in cases after EVAR or in bypass graft occlusions include surgery and endovascular therapies like thrombectomy, thrombolysis, angioplasty, and stenting to prevent further occlusion. Some open methods for iliac limb graft occlusion include surgical thrombectomy with a Fogarty catheter or with Vollmar ring stripper. However, these techniques pose risks of stent dislocation, disruption of sealing zones, or recurrences. Although the Vollmar ring stripper method has shown some success compared to the Fogarty catheter, there are still limitations [2]. In cases of failure with conventional methods, additional bypass implantation, such as femoro-femoral crossover bypasses or axillofemoral bypasses, may be necessary. However, these procedures have low primary patency rates.

In an interesting case series by Xiaofeng Han et al., 2021, and Arindam Chaudhuri et al., 2023, the usage of aspirational thrombectomy as well as rotational thrombectomy devices in treating iliac limb graft occlusion (LGO) post-EVAR was discussed [3,4]. Xiaofeng Han et al., 2021, used the AngioJet Ultra thrombectomy device in 12 patients, and the procedure was performed percutaneously. After percutaneous mechanical thrombectomy (PMT) with AngioJet™ and additional balloon dilation angioplasty, a bifurcated endograft was implanted in 9 patients for relining. In the remaining 3 patients, they implanted unibody endografts. This treatment regime maintained the patency of limbs for at least 12 months. Ongoing studies will provide new real-life data in a large and unselected patient population to better understand the unibody device’s advantages and limitations.

Arindam Chaudhuri et al., 2023, performed a percutaneous mechanical thrombectomy with a 10F Rotarex™ (BD, Franklin Lakes, NJ, USA) Rotational Atherectomy System followed by additional limb stenting and limb relining in all of the 6 cases mentioned in the series. Out of the 6 cases, 4 patients had undergone EVAR, 1 patient had undergone fenestrated EVAR with an iliac branch device, and 1 patient had undergone branched EVAR. Post-procedural outcomes showed, according to the study, a short-to-midterm patency of the iliac limbs.

However, the limitations of additional relining procedures are associated with the risk of reinterventions, lumen loss, and endoleaks. Relining does not reinforce the fixation and seal capacity of the original EVAR grafts. In the percutaneous methods using large-bore catheters, one has to use vascular closure devices, which again pose a threat of mechanical obstruction, hematoma, pseudoaneurysm formation.

Acute and subacute leg ischemia in bypass occlusion poses limb survival risks, warranting prompt intervention. Percutaneous mechanical thrombectomy, including rotational thrombectomy using Rotarex™ (BD, Franklin Lakes, NJ, USA), is a recent option, alongside CDT (catheter-directed thrombolysis) and surgical thrombectomy. Surgical thrombectomy with the Fogarty catheter shows increased perioperative complications but limited technical success. Local lysis incurs higher costs due to intensive monitoring and repeat angiographies, plus bleeding risks. Failure to recanalize the bypass graft leads to redo-bypasses or implantation of long infragunial bypass with poor primary as well as secondary patency rates. In the worst-case scenario, the failure would lead to irreversible ischemia and limb amputation.

The AngioJet™ Ultra Thrombectomy System (Boston Scientific, Marlborough, MA, USA) is a pharmacomechanical aspiration thrombectomy device with multiple catheter options. According to the literature, it can be utilized in a wide range of vessels, from a minimum diameter of 1.5 mm to large vessels. The Solent™ Proxi catheter is suitable for vessels with smaller diameters (3 mm), accessible with ipsilateral access, while the ZelanteDVT™ 8F catheter is suitable for wide-bore vessels or vascular conduits (diameter ≥ 6 mm). Both catheters require 0.035-inch guide wires as standard.

In cases of persistent thrombus load, local lysis can be performed using pulse spray technology. Studies using the AngioJet™ Thrombectomy device on native tibial vessels have shown a good success rate [5]. Other forms of rotational MT, such as Rotarex™ (BD, Franklin Lakes, NJ, USA) [6]., have been used on vascular conduits with proven success, achieving primary and secondary patency rates of 66% and 86%, respectively [7].

Aspirational thrombectomy can be used as either an individual primary intervention or as an adjuvant to traditional surgical methods in hybrid interventions and emerges as a viable alternative to redo bypass surgery in cases of graft failure, preserving native vessels for further, more intensive surgical revascularization methods.

## 5. Conclusions

A hybrid approach combining surgical thrombectomy with aspiration thrombectomy using the AngioJet™ Ultra Thrombectomy System has proven highly successful in the immediate, short-term, as well as medium-term postoperative phases. This combination not only achieved limb salvage in both cases but also reduced procedural time in chronically ill and debilitated patients (ASA III & IV). Furthermore, patient with wounds in the early phase showed a dramatic positive response and aided in healing. Both patients were postoperatively advised to continue anticoagulation therapy with SAPT plus low-dose DOAC for at least 6 months. Upon further controlled duplex sonographic examination and assessment of the flow and intimal growth pattern of the vessels at regular intervals, we would reevaluate the anticoagulation.

## Data Availability

Data are contained within the article.
